# New York Letter

**Published:** 1880-10

**Authors:** 


### Domestic Correspondence.

---

ARTICLE VI.

### NEW YORK LETTER.

*Messrs. Editors* :—There is a young man over in Brooklyn by the name of Livingstone, who is trying to outdo Tanner. He claims to have fasted before, and to have shown his physical endurance by walking some thousands of quarter miles in an equal number of quarter hours. He has not as yet attracted the attention of the public; neither has the medical profession shown any interest in his performance, although the opportunity to get a name in the papers is too good to be long neglected. But the people are tired of Tanner and his ways; and no amount of exhibitions will ever make one meal a day popular in New York. The once immaculate Tanner, by the way, went into a grocery store last week and weighed himself. He tipped the scales at 157 pounds, which is only half a pound less than before his fast. Finding himself so thoroughly recovered, he advertised a lecture in Booth's theatre on "What I know about Fasting." He was introduced to a thin audience by a medical Eclectic light of the name of Gunn. Dr. Tanner then delivered himself of some choice diatribes against all doctors in general, and regular doctors in particular. He would have all his audience give up medicine forever and recuperate on oxygen, water and animal electricity. Dr. Tanner, I should judge, has much vituperative power and a very lively scientific imagination, and neither the absence of solid food or of professional sympathy can curtail his infinite vivacity. It is much to be hoped that he will now marry Miss Mollie Fancher, the fasting girl, and go into a trance.

The last hot wave left our city a week ago, and the cool breezes are now bringing back our doctors and their patients. New York seems to empty itself into the country more and more every summer; and at the same time to have more people than ever to stay and take their chances with the sun. By August, it may be a decided misfortune if one who remains in the city falls suddenly in need of a doctor. A case in hand has just been told me. Dr. A went out of the city and left his patients with Dr. B.; Dr. B left his with Dr. C, and Dr. C was suddenly called away and left the whole lot to the gentle mercy of nobody in particular. A patient of Dr. A's was taken ill; she sent around to A and B and C; of course to no purpose. She then sent for a fourth physician, who was also out of town, and finally to a fifth who sent back word that he was just on his way to catch the train and could not call. By this time the lady was quite comfortable and concluded to get along without a doctor.

The mortality in New York has, like the heat, been something extraordinary. The ordinary mortality rate of the city is a pretty high one, being from 25 to 28 per 1,000. In the week ending June 12, it was 25.13; then it began to rise. In the week ending June 26, it was 44.97, while the reported mortality for the week ending July 3, was 56.20 per 1,000. This is a very much larger rate than occurs in any other city in this country or abroad, so far as statistical returns show. There were during that week 1,297 deaths, of which two-thirds were of children under the age of five years, and one-half, of children under the age of one year. About two-thirds of the children that died lived in the tenement houses. But it is a somewhat singular fact that the proportion of deaths in these houses was not greatly changed. That is to say, about two-thirds of the deaths in the city occur in the tenement houses all the year round whether it is hot or cold. It shows that the great cause of the deaths is the intense heat, and that a baby is not much safer from it up on Fifth Avenue than down in the slums about Mulberry Street. A great deal is done by the city and by charitable people to give the children relief, and the sea-side sanitaria, the free excursions into the country and upon the water, are among the best known and most useful of New York's many charities.

We are just now having a few innovations. The city has established a night medical service, so that strangers or any one needing immediate medical help can be at once supplied. The names of about 300 physicians have been sent in to the Police Board. These have been divided into smaller lists each one including all the physicians living in a single precinct. The lists are posted in the various police stations and if a person is taken suddenly sick in the night he sends them for a doctor. The fee for a night call of this kind is $3. In Paris, where the service originated, and where it is said to have proved useful, there are, I believe, ten or twenty calls every night. This is not a very large number for two millions of people, and New York cannot expect so many even as this. The new service is not likely to enrich the physician very much, and it is very doubtful whether humanity demanded it. New York is pretty well peppered with physicians everywhere, and most of them are willing enough to respond to night-calls. Nobody can go to a police station (provided he knows where it is—which few do) without passing half a dozen doctors' signs at least. The New York Night Medical Service has a humane and sonorous sound, but it appears very much like a piece of supererogation. However, it may succeed, in which case Chicago can benefit by the example.

Another little performance, which we are now carrying on, is that of registration. The work of signing the name, taking an oath, and paying from twenty-five to seventy-five cents, according to each doctor's special gullibility in the hands of the county clerk, is proceeding very briskly. It must be finished by October 1, but there are still 1,500 or more doctors to register and only two weeks to do it in. There must be some left over; and then there will be a wail over the $20 fine incurred. The measure is a popular one among regular physicians, as it obliges every man who hangs out his sign as a doctor to show his authority for doing so. This he does by swearing that he has a diploma or license from a reputable college or chartered medical society. Of course anybody can swear to this, and it must depend on others, who are on the watch, to find him out. We expect our county medical society to do the work here, and I have a great deal of confidence that they will do it well. There are some very active and wide-awake men on the censorship committee. If the city officials stand by the law, it is to be expected that there will be lively times among the charlatans, abortionists, magnetic doctors, etc., of the city. The Homœopathic Medical College has, it is rumored, done a very magnanimous thing. The new law provides that hereafter any person who comes into the State to practice must first have his diploma or license examined and endorsed by some regularly incorporated medical college in the State. For this indorsement the sum of $20 is to be paid. The Homœopathic Medical College offers to furnish this indorsement for nothing! Whether this is pure benevolence, a desire to advertise, or an ambition to increase the adherents of homœopathy in the State I dare not say.

I wrote sometime ago about a novel rhinoplastic operation which Dr. Sabine performed upon a patient at Bellevue Hospital. The boy had lost his nose, and to replace it, the middle finger of the left hand was sewed on and then amputated from the hand. I have seen the boy since. He is still far from handsome, but the finger stays on and the general health is good. The tip of the finger failed to unite, however, and after the amputation there was some suppuration and part of the first phalanx necrosed and came away. So that the end of the new organ lacks something of the rounded symmetry of nature. And as a whole it has a little of a foreign air; it does not seem to feel thoroughly at home in its new surroundings. But "Tommy," the patient, is pleased and has confidence in the nose of the future and the efficacy of future operations. It is intended to sew up the top of the nose again, to construct some nostrils and add various other little touches in the interests of plastic surgery, high art and of a most exemplary patient.

The colleges are beginning their annual courses and medical students are pouring into town, gladdening the heart of the boarding-house keeper. Bellevue Hospital Medical College began its regular term Sept. 15, and it contiues for six months. The University Medical College begins its preliminary course on the 15, and its regular course Oct. 1. This is the only one of the three large colleges which has shown no regard to the now general demand for longer annual courses, and more of them.

It sticks to its old system, and perhaps deservedly, has the reputation of sending out the biggest and the worst medical classes every year. But it will have to yield bye and bye. By chance, I have learned of several country physicians who have heretofore been in the habit of sending their pupils to the university. They now intend to make a change and send them elsewhere. The college has excellent instructors, and is as well equipped as its rivals; but it ought to show a higher ambition than that of graduating the largest class of doctors in the city. The College of Physicians and Surgeons has done something in the line of higher education. Its annual course is now seven months, instead of five, lasting from October 1st into May. It recommends in italics that all students attend three courses of lectures, but it has not the moral courage to compel the plan, and its adoption of a long course rather indicates that it does not intend to do anything of the kind.

There has sprung up in the last three or four years something like a new industry, in the line of medical education. It is the system of "cram-quizes." For many years there have been two or three physicians—oftener a less number—who have taken students intending to compete for the positions in the hospitals, and have put them through a six months' "cram," working them at the highest possible pressure. This method turned out, at the end of the half year, young men so thoroughly informed in every point, so ready and well drilled that the ordinary student had no chance against them. It gradually became a necessity, in order to get a hospital position, to go through a "cram-quiz." The fee for half-a-year's cramming was $100; and if the quiz-master had a dozen or two pupils, as he generally did, the profit was a very respectable, though a well-earned one. Quizzing having thus become profitable, has now become very popular. Where there were only two or three notices on the bulletin-boards, there are now a dozen. The number of pupils has largely increased also. This is partly because there are more hospital positions than formerly, and partly because many of the quiz-masters take students and cram them for the college examinations only. The quiz system is, of course, liable to abuse, and occasionally a student gets permanently injured by the strain it puts upon him. They often cause a neglect of the didactic lectures also, which is not, however, always a misfortune. But whatever the danger of abuse, it cannot be doubted that there is nothing which compels study, trains the attention, and disciplines the memory like a cram-quiz, if, as is generally the case, the preceptor is alive and the pupils are ambitious. It is a fact useful to be noted, in the cause of higher education, that in the competitions for the hospitals, competitions in which the best men of all the colleges enter, those who have had a previous academic training almost uniformly come out ahead. Five-sixths of the house-staffs on the large city hospitals are bachelors or masters of arts. In these days, when the practical value of a classical education is so often doubted, I for one am glad to furnish a little evidence in its favor.

NEW YORK, Sept. 15, 1880.

---

*Le Journal d'Hygiène*, according to the *Revue Médicale Française et Etrangère*, reports three cases of absolute but temporary impotence arising from the use of salicylate of sodium, the dose being only from three to four grams in the twenty-four hours.

Says the *Journal d'Hygiene:* "These attacks on virility are the more important as without our knowledge we are daily taking the salicylate of sodium in our food and drinks, as in the butter, beer, wine, preserves, and that a man may thus unconsciously do a serious injury to his wife. If the fact is confirmed, the salicylate of sodium will become a source of disorder in the family."

DR. MARY J. MERGLER has returned to the city, after a year's study at the University of Zurich, and has entered upon the practice of her profession. She has also resumed her work at the Woman's Medical College, as Assistant to the Chair of Materia Medica and Therapeutics. The valuable quality of her work in the field of histology has been appreciated in this city.

